# *In Vitro* Effects of the Endocrine Disruptor *p,p*’-DDT on Human Follitropin Receptor

**DOI:** 10.1289/ehp.1510006

**Published:** 2016-02-19

**Authors:** Mathilde Munier, Julie Grouleff, Louis Gourdin, Mathilde Fauchard, Vanessa Chantreau, Daniel Henrion, Régis Coutant, Birgit Schiøtt, Marie Chabbert, Patrice Rodien

**Affiliations:** 1 MITOVASC Institute, Angers, France; 2 UMR CNRS 6214, INSERM 1083, Laboratory of Integrated Neurovascular and Mitochondrial Biology, University of Angers, Angers, France; 3 Reference center for rare diseases of hormonal receptivity, Angers, France; 4 Department of Endocrinology, University Hospital, Angers, France; 5 Interdisciplinary Nanoscience Center,; 6 Center for Insoluble Protein Structures, and; 7 Department of Chemistry, Aarhus University, Aarhus, Denmark; 8 Department of Pediatric Endocrinology, University Hospital, Angers, France

## Abstract

**Background:**

1-chloro-4-[2,2,2-trichloro-1-(4-chlorophenyl)ethyl]benzene (p,p′-DDT) is a persistent environmental endocrine disruptor (ED). Several studies have shown an association between p,p′-DDT exposure and reproductive abnormalities.

**Objectives:**

To investigate the putative effects of p,p′-DDT on the human follitropin receptor (FSHR) function.

**Methods:**

and Results: We used Chinese hamster ovary (CHO) cells stably expressing human FSHR to investigate the impact of p,p′-DDT on FSHR activity and its interaction with the receptor. At a concentration of 5 μM p,p′-DDT increased the maximum response of the FSHR to follitropin by 32 ± 7.45%. However, 5 μM p,p′-DDT decreased the basal activity and did not influence the maximal response of the closely related LH/hCG receptor to human chorionic gonadotropin (hCG). The potentiating effect of p,p′-DDT was specific for the FSHR. Moreover, in cells that did not express FSHR, p,p′-DDT had no effect on cAMP response. Thus, the potentiating effect of p,p′-DDT was dependent on the FSHR. In addition, p,p′-DDT increased the sensitivity of FSHR to hCG and to a low molecular weight agonist of the FSHR, 3-((5methyl)-2-(4-benzyloxy-phenyl)-5-{[2-[3-ethoxy-4-methoxy-phenyl)-ethylcarbamoyl]-methyl}-4-oxo-thiazolidin-3-yl)-benzamide (16a). Basal activity in response to p,p′-DDT and potentiation of the FSHR response to FSH by p,p′-DDT varied among FSHR mutants with altered transmembrane domains (TMDs), consistent with an effect of p,p′-DDT via TMD binding. This finding was corroborated by the results of simultaneously docking p,p′-DDT and 16a into the FSHR transmembrane bundle.

**Conclusion:**

p,p′-DDT acted as a positive allosteric modulator of the FSHR in our experimental model. These findings suggest that G protein–coupled receptors are additional targets of endocrine disruptors.

**Citation:**

Munier M, Grouleff J, Gourdin L, Fauchard M, Chantreau V, Henrion D, Coutant R, Schiøtt B, Chabbert M, Rodien P. 2016. In vitro effects of the endocrine disruptor p,p′-DDT on human follitropin receptor. Environ Health Perspect 124:991–999; http://dx.doi.org/10.1289/ehp.1510006

## Introduction

The health impact of endocrine disruptors (EDs) is of growing concern because their targets and effects on animals and humans are diverse, and the list of disruptors seems endless (Zoeller et al. 2012). Among EDs, dichlorodiphenyltrichloroethane (DDT), an organochlorine pesticide composed mainly of 1-chloro-4-[2,2,2-trichloro-1-(4-chlorophenyl)ethyl]benzene (*p,p*′-DDT), was largely used after the Second World War for its insecticidal properties. Although *p,p*′-DDT was banned in the 1970s in the Western world, it continues to be used in developing countries. It is known to accumulate in fatty tissue, and it is highly persistent in the environment. Contamination of soil and water allows *p,p*′-DDT to ascend the food chain and to reach humans (Sudharshan et al. 2012). Children are exposed to maternal *p,p*′-DDT *in utero* and through breast feeding. For example, the average serum concentration of *p,p*′-DDT approaches 4 ng/g (7.3 × 10^–11^ M) of body lipids in the French population (Saoudi et al. 2014). However, in a population of young men in South Africa, where DDT continues to be sprayed, the average lipid-adjusted serum concentration of *p,p*′-DDT reached 90.23 μg/g (1.5 × 10^–6^ M) (Aneck-Hahn et al. 2007). According to epidemiological data, exposure to *p,p*′-DDT is associated with decreased semen parameters (Jeng 2014; Martenies and Perry 2013). Moreover, cryptorchidism, hypospadias, and micropenis have been reported to be associated with *in utero* exposure to *p,p*′-DDT (Damgaard et al. 2006; Gaspari et al. 2012; Hosie et al. 2000; Jeng 2014; Rignell-Hydbom et al. 2012), and the concept of testicular dysgenesis syndrome has been proposed to encompass the spectrum of male reproductive outcomes that have been associated with ED exposure (Wohlfahrt-Veje et al. 2009). In addition, *p,p*′-DDT has been measured in the ovarian follicular fluids of women (Jarrell et al. 1993; Jirsová et al. 2010), and *p,p*′-DDT exposures have been associated with evidence of reduced fertility (Jirsová et al. 2010; Venners et al. 2005). Shortened menstrual cycles (Windham et al. 2005) and a reduced probability of pregnancy in daughters of women exposed to *p,p*′-DDT (Cohn et al. 2003) have been reported. Moreover, serum *p,p*′-DDT and *in utero* exposure have been associated with precocious puberty in girls (Ouyang et al. 2005; Vasiliu et al. 2004). Some *in vitro* studies have shown that *p,p*′-DDT exhibits anti-androgenic and estrogen-like effects (Aubé et al. 2011; Kojima et al. 2004; Li et al. 2008; Schug et al. 2011; Strong et al. 2015; Wang et al. 2010) through binding to nuclear receptors. Gonadal function is under pituitary control *via* the gonadotropin hormones: follicle stimulating hormone (FSH) and luteinizing hormone (LH). A third hormone, human chorionic gonadotropin (hCG), is secreted by the placenta and controls ovarian function during gestation in primates.

The FSH receptor (FSHR) is a plasma membrane receptor that, along with the LH/hCG receptor, belongs to the G protein–coupled receptor (GPCR) superfamily (Minegishi et al. 1991). FSHR is expressed in Sertoli and granulosa cells in male and female gonads, respectively, and is required for normal spermatogenesis and growth and maturation of ovarian follicles, as well as for estrogen production (Siegel et al. 2013). It is mainly coupled to the cAMP pathway through the G_sα_ subunit and adenylyl cyclase (AC) (Means et al. 1974; Minegishi et al. 1994). However, it can also couple to several other effectors such as G_αq_ and β-arrestin (Gloaguen 2011; Landomiel et al. 2014; Ulloa-Aguirre et al. 2007). Previously, *p,p*′-DDT has been shown to disturb the downstream signaling of the FSHR (Bernard et al. 2007; Rossi et al. 2007), and *p,p*′-DDE, a metabolite of *p,p*′-DDT, increased FSH-induced progesterone production (Crellin et al. 1999) and aromatase activity (Younglai et al. 2004) in porcine and human granulosa cells, respectively.

Although FSH interacts with the large extracellular N-terminal domain of its receptor, small molecules have been designed that can activate or inhibit the FSHR (Arey et al. 2008; Dias et al. 2011, 2014; Sriraman et al. 2014; van Koppen et al. 2013; Wrobel et al. 2006; Yanofsky et al. 2006; Yu et al. 2014). These molecules bind to the transmembrane domain (TMD) of the FSHR and can be considered to be allosteric modulators. *p,p*′-DDT shows structural homologies with some of the allosteric modulators of FSHR (Dias et al. 2011; van Koppen et al. 2013). This suggests that *p,p*′-DDT may interact with allosteric sites on the FSHR. We investigated the effects of *p,p*′-DDT in Chinese hamster ovary (CHO) cells stably transfected with human FSHR (CHO-FSHR) and responsive to FSH. We showed that *p,p*′-DDT increased the cAMP response to FSH through an interaction with the TMD of FSHR, providing evidence for an allosteric effect of *p,p*′-DDT on this receptor.

## Materials and Methods

### Reagents

Chemicals: *p,p*′-DDT, forskolin, 3-isobutyl-1-methylxanthine (IBMX), salmon calcitonin, 1-chloro-4-[2,2-dichloro-1-(4-chlorophenyl)ethenyl]benzene (*p,p*′-DDE), 1-chloro-2-[2,2,2-trichloro-1-(4-chlorophenyl)ethyl]benzene (*o,p*′-DDT), and bisphenol A (BPA) were purchased from Sigma-Aldrich and dissolved in dimethyl sulfoxide (DMSO). The gonadotropin hormones hFSH (Gonal-f) and hCG (Ovitrelle) were purchased from Merck-Serono. The conversion between international units per milliliter and nanograms per milliliter or molar concentrations is as follows: 1 IU/mL recombinant hFSH corresponds to 100 ng/mL or 3.3 nM, and 1 IU/mL recombinant hCG corresponds to 62 ng/mL or 2 nM.

Plasmids: FSHR mutants T3.32A, T3.32I, H7.42A, T3.32I-H7.42A, and rat FSHR were kindly provided by S. Costagliola [IRIBHM (Institute of Interdisciplinary Research in Molecular Human Biology), Université Libre de Bruxelles, Belgium]. Amino acid residues are numbered according to the Ballesteros system (Sealfon et al. 1995).

### Cell Culture

CHO cell lines stably transfected with human FSHR have been described previously (Bonomi et al. 2006). CHO and CHO-FSHR cell lines were maintained in Dulbecco’s modified Eagle’s Medium (DMEM, PAA) containing 10% fetal calf serum (FCS, Biowest), 2 mM glutamine, 100 U/mL penicillin, and 100 μg/mL streptomycin (Lonza) at 37°C in a humidified incubator gassed with 5% CO_2_.

### cAMP Assay

cAMP production was determined using the Promega GloSensor cAMP assay (Promega) (Binkowski et al. 2011). Briefly, cells were seeded (20,000 cells/well) in white 96-well clear-bottomed microplates. The next day, the cells were transfected with pGloSensorTM-22F cAMP plasmid (150 ng) encoding an engineered cAMP-sensitive luciferase, using Lipofectamine LTX (Invitrogen, Cergy-Pontoise, France) according to the manufacturer’s instructions. Twenty-four hours after transfection, the medium was removed, and the cells were incubated for 2 hr at 20°C in 90 μL of the equilibration medium, a substrate-containing medium (GloSensor^TM^ cAMP reagent) diluted to 6% in DMEM containing 10% FCS. The cells were incubated with *p,p*′-DDT, *p,p*′-DDE, or hormones for 30 min, and end-point luminescence was recorded on a Synergy^TM^ 2 microplate luminometer (Biotek). Graphs were fitted to the data using GraphPad Prism 6 (GraphPad Software, Inc.), and the results are expressed as the mean ± SEM from at least three independent experiments performed in triplicate. Concentration–response data were fitted using a four-parameter equation.

### Molecular Modeling and Induced Fit Docking

FSHR was modeled from I1.29 to S7.69 with MODELLER 9v8 (Sali and Blundell 1993) by homology with rhodopsin (PDB code 3C9L), except for TM5, which was modeled as a straight helix (Kleinau et al. 2011). The FSHR model was prepared for docking using the Protein Preparation Wizard in Schrödinger Suite 2012 (Schrödinger Suite 2012 Protein Preparation Wizard; Epik v.2.3, Impact v.5.8, Prime v.3.1). Protonation states were assigned for all titrable groups according to pH 7 using Propka (Olsson et al. 2011), and the model was then energy minimized using the OPLS2005 force field with a restraint in which the maximum heavy atom root mean square deviation (RMSD) was set to 0.30 Å. The induced fit dockings (IFDs) (Sherman et al. 2006a, 2006b) were performed in Schrödinger Suite 2012 (Schrödinger Suite 2012 Induced Fit Docking Protocol; Glide v.5.8; Prime v.3.1) according to a three-step protocol: *a*) the initial Glide docking was performed with 0.5 scaling of all van der Waals radii for a maximum of 50 poses; *b*) side chains of residues within 5 Å of the ligand were optimized, with an implicit membrane model; *c*) a final Glide docking was performed for complexes that were within 30 kcal/mol of the best scoring complex and within the top 20 overall. *p,p*-DDT was docked into the minor pocket (Hoyer et al. 2013) (TM1-3,7). The pose with the best IFD score was then used as input for an IFD calculation for 3-((5methyl)-2-(4-benzyloxy-phenyl)-5-{[2-[3-ethoxy-4-methoxy-phenyl)-ethylcarbamoyl]-methyl}-4-oxo-thiazolidin-3-yl)-benzamid (16a) in the major site. Additionally, 16a was docked into the major pocket (TM3-7) with the minor site unoccupied, and the highest-scoring pose was used as input in an IFD calculation for *p,p*′-DDT in the minor site. The reverse procedure, docking of 16a in the major pocket followed by binding of *p,p*′-DDT in the minor pocket, led to similar results to those obtained by first binding *p,p*′-DDT and then binding 16a (data not shown).

### Statistical Analyses

Results represent the mean ± SEM of at least nine samples, obtained in at least three independent experiments for each condition. Statistical analyses were performed using the nonparametric Mann–Whitney test (Prism 6, GraphPad Software, Inc.).

## Results

### Effects of *p,p*′-DDT on FSH-Dependent cAMP Production

**Figure 1:**
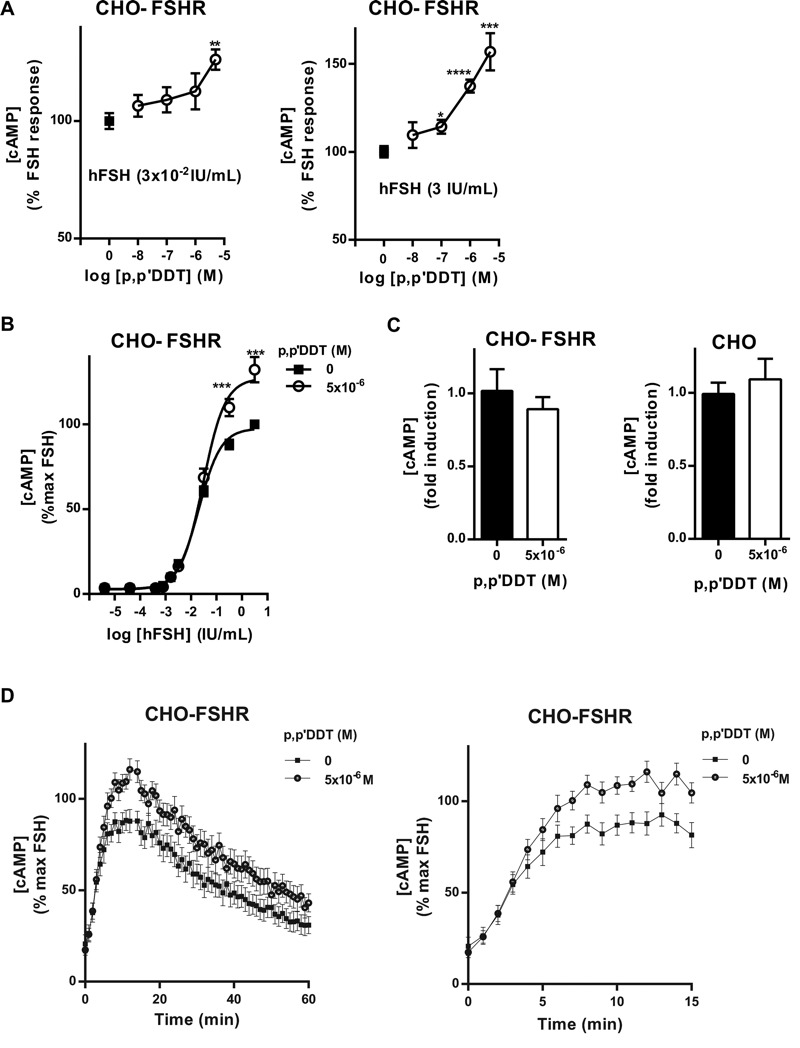
Effects of *p,p*′‑DDT on follitropin (FSH)-stimulated cAMP production. (*A*) Chinese hamster ovary-FSH receptor (CHO-FSHR) cells were incubated with hFSH at 3 **×** 10^–2^ IU/mL and 3 IU/mL and increasing concentrations of *p,p*′‑DDT were investigated (means ± SEM of four independent experiments performed in triplicate). The cAMP concentration measured in the presence of hFSH alone was arbitrarily set at 100%, and the differences were evaluated using the Mann–Whitney U test. (*B*) Dose–response curve of hFSH on CHO-FSHR cells with or without *p,p*′‑DDT (5 **×** 10^–6^ M) (means ± SEM of eight independent experiments performed in triplicate). The maximum response to FSH was arbitrarily set at 100%, and the differences were evaluated using a two-way analysis of variance (ANOVA). (*C*) Basal cAMP production of CHO-FSHR and CHO treated with *p,p*′‑DDT (5 **×** 10^–6^ M) (means ± SEM of four independent experiments performed in triplicate). The basal cAMP level in the absence of *p,p*′‑DDT was arbitrarily set at 1. (*D*) Cells were stimulated with 3 IU/mL hFSH in the presence of *p,p*′‑DDT (5 **×** 10^–6^ M). The luminescence was recorded every minute (means ± SEM of five independent experiments performed in triplicate). The maximum response to FSH was arbitrarily set at 100%. For clarity, the curve depicting the early phase of the kinetics is enlarged on the right. *p < 0.05, **p < 0.01, ***p < 0.001, ****p < 0.0001 for the response in *p,p*′‑DDT–exposed compared with unexposed cells.

To investigate the effects of *p,p*′-DDT on FSHR, we used CHO cells that were stably transfected with human FSHR (CHO-FSHR) (Bonomi et al. 2006). We first verified that 5 × 10^–6^ M *p,p*′-DDT did not induce cell death (see Figure S1). The dose–response curve for hFSH in these cells indicated an EC_50_ value of 0.03 ± 0.002 IU/mL (data not shown). *p,p*′-DDT enhanced the cAMP accumulation induced by two different doses of hFSH, 0.03 IU/mL or 3 IU/mL, in coincubation (Figure 1A) up to 157 ± 10.57% of the maximum response. We next examined the effect of the most potent concentration of *p,p*′-DDT (5 × 10^–6^ M) on the FSH dose–response curve. The maximum response was increased by 32 ± 7.45% (eight experiments), whereas the EC_50_ was unaffected (0.02 IU/mL vs. 0.03 IU/mL) (Figure 1B). In contrast to the increase of the maximum response, there was no impact on the basal activity of the FSHR (Figure 1C). In the kinetic study, the effects of *p,p*′-DDT were detected as early as 6 min (Figure 1D), whereas the maximum response to FSH with and without *p,p*′-DDT was reached at 13 min and 12 min, respectively (Figure 1D).

### Effects of *p,p*′-DDT on Other Receptors

**Figure 2:**
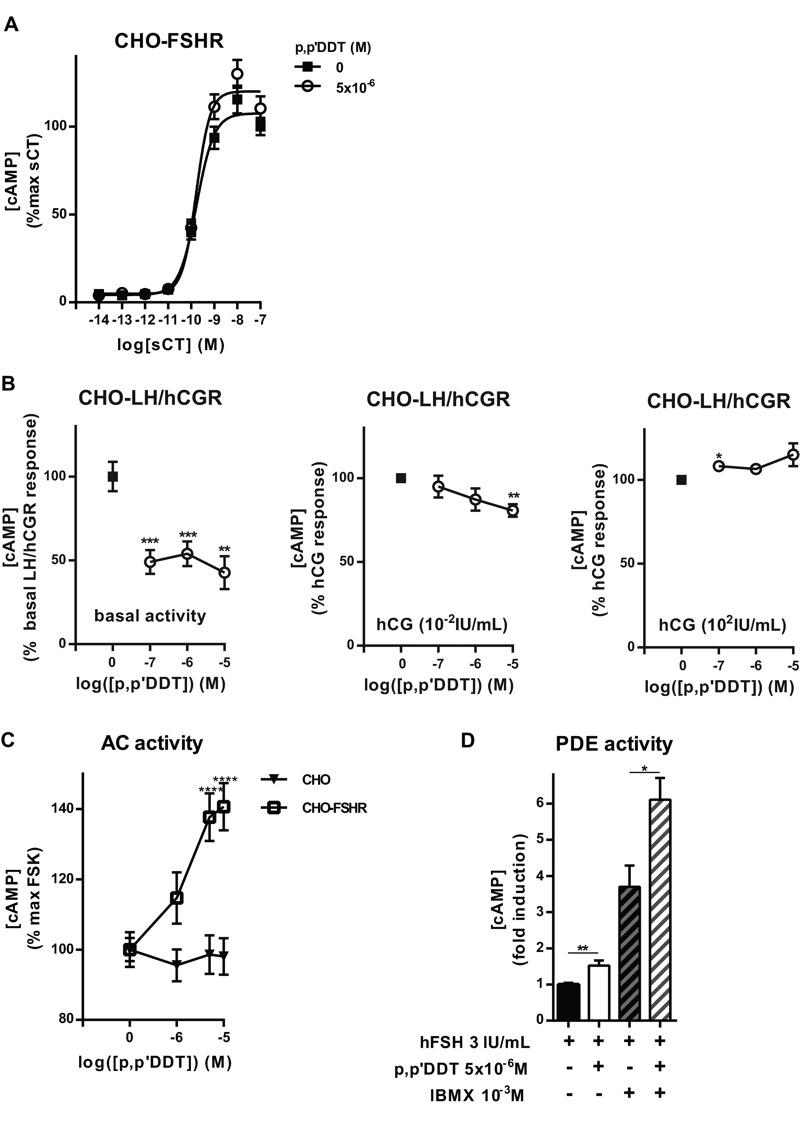
Effects of *p,p*′‑DDT on calcitonin-, human chorionic gonadotropin (hCG)-, and forskolin (FSK)-stimulated cAMP production and on inhibition of phosphodiesterase (PDE) by IBMX. (*A*) Chinese hamster ovary–follitropin receptor (CHO-FSHR) cells were stimulated for 30 min with increasing concentrations of salmon calcitonin (sCT) with or without 5 **×** 10^–6^ M *p,p*′‑DDT (means ± SEM of three independent experiments performed in triplicate). The maximum response to sCT alone was arbitrarily set at 100. (*B*) Basal and hCG-stimulated (hCG 10^–2^ IU/mL and 100 IU/mL) cAMP production was measured in CHO-luteinizing hormone/hCGreceptor (CHO-LH/hCGR) cells with or without *p,p*′‑DDT (means ± SEM of three independent experiments performed in triplicate). The cAMP production in the absence of *p,p*′‑DDT was arbitrarily set at 100, and the differences were evaluated using the Mann–Whitney U test. (*C*) CHO-FSHR and CHO cells were stimulated with 10^–5^ M forskolin [an adenylate cyclase (AC) agonist] and increasing doses of *p,p*′‑DDT (means ± SEM of three independent experiments performed in triplicate). The cAMP production in the presence of forskolin alone was arbitrarily set at 100, and the differences were evaluated using the Mann–Whitney U test. (*D*) CHO-FSHR cells were incubated with or without 1 mM IBMX for 2 hr and then stimulated or not with FSH 3 IU/mL with or without *p,p*′‑DDT 5 **×** 10^–6^ M (means ± SEM of three independent experiments performed in triplicate). The cAMP production in the presence of FSH alone was arbitrarily set at 1, and the differences were evaluated using the Mann–Whitney U test. *p < 0.05, **p < 0.01, ***p < 0.001 for the response in *p,p*′–DDT-exposed compared with unexposed cells.

In CHO-FSHR cells, cAMP production in response to calcitonin stimulation of the endogenously expressed calcitonin receptor was not affected by coincubation with *p,p*′-DDT (Figure 2A). In addition, *p,p*′-DDT did not induce cAMP in response to calcitonin in CHO cells (data not shown).

The effects of *p,p*′-DDT on the LH/hCG receptor (LH/hCGR), a closely related receptor that belongs to the same family as the FSHR (Vassart et al. 2004), were also analyzed. In CHO cell lines stably transfected with the human LH/hCGR (CHO-LH/hCGR) (Bonomi et al. 2006), *p,p*′-DDT decreased the cAMP production stimulated by hCG at a concentration of 0.01 IU/mL, in a dose-dependent manner, to 80 ± 3.7% of the response in the absence of *p,p*′-DDT (Figure 2B). The response to 100 IU/mL hCG was also increased in response to *p,p*′-DDT, but the increase was significant only at the lowest dose of *p,p*′-DDT (10^–7^ M) (Figure 2B). Interestingly, *p,p*′-DDT decreased the basal activity of LH/hCGR by 50 ± 9%.

To examine the putative impact of *p,p*′-DDT on the downstream effectors of the FSHR, we first tested its effects on forskolin-induced cAMP accumulation in CHO-FSHR and CHO cells (Figure 2C). There was a dose-dependent increase of the response to forskolin in CHO-FSHR cells that reached 140 ± 6.71% of the control value. This effect was not observed in CHO cells (Figure 2C). In addition, we did not observe an effect of *p,p*′-DDT on the response to forskolin in HEK293 cells or in the CHO-LH/hCGR cells (data not shown). These findings suggest that the effects of *p,p*′-DDT on AC require the presence of the FSHR. We also analyzed the effects of *p,p*′-DDT on phosphodiesterase (PDE) activity. *p,p*′-DDT further increased the already-elevated FSH-stimulated cAMP production observed in the presence of IBMX, a PDE inhibitor (Figure 2D).

### Interactions Between *p,p*′-DDT and the FSH Receptor Transmembrane Domain

**Figure 3:**
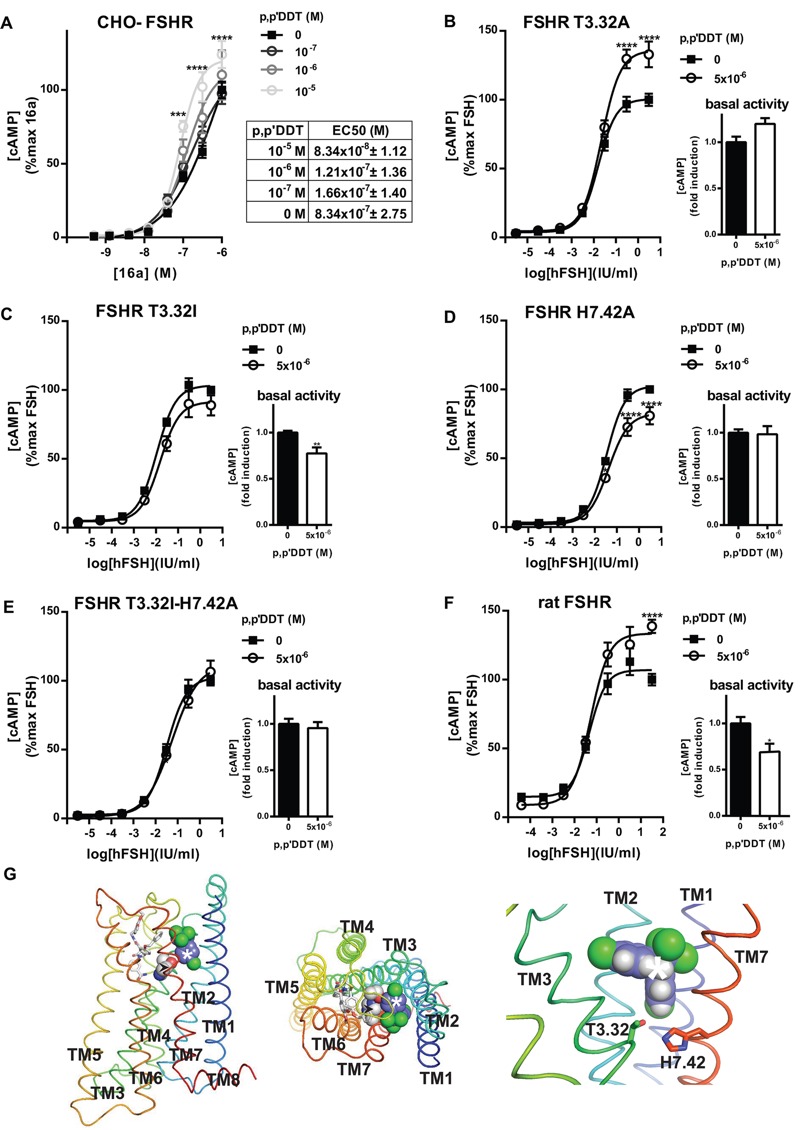
*p,p*′‑DDT targets the follicle stimulating hormone receptor (FSHR) transmembrane domain. (*A*) Chinese hamster ovary–follitropin receptor (CHO-FSHR) cells were stimulated for 30 min by increasing doses of 3-((5methyl)-2-(4-benzyloxy-phenyl)-5-{[2-[3-ethoxy-4-methoxy-phenyl)-ethylcarbamoyl]-methyl}-4-oxo-thiazolidin-3-yl)-benzamid (16a) in the presence of increasing concentrations of *p,p*′‑DDT (means ± SEM of six independent experiments performed in triplicate). The maximum response to 16a was arbitrarily set at 100, and the differences were evaluated using the Mann–Whitney U test. (*B–F*) Effects of *p,p*′‑DDT on mutant FSHR T3.32A (*B*), T3.32I (*C*), H7.42A (*D*), T3.32I-H7.42A (*E*) and rat FSHR (*F*) transiently expressed in CHO cells and stimulated for 30 min with increasing concentrations of FSH with or without *p,p*′‑DDT (means ± SEM of three independent experiments performed in triplicate). The maximum response to hFSH in the absence of *p,p*′‑DDT was arbitrarily set at 100. The basal activity measured in absence of FSH with (white columns) or without (black columns) *p,p*′‑DDT. The basal activity in absence of *p,p*′‑DDT was arbitrarily set at 1, and the differences were evaluated using the Mann–Whitney U test. (*G*) Side and top views of the putative binding mode of *p,p*′‑DDT and 16a in the transmembrane domain (TMD) of FSHR. *p,p*′‑DDT is shown as spheres [carbon (C), purple; chlorine (Cl), green; hydrogen (H), gray], and 16a is shown as sticks [C, white; nitrogen (N), blue; oxygen (O), red]. FSHR is shown as a ribbon representation. The helices are colored from blue for TM1 to red for TM7 and the intracellular TM8. Thr3.32 and His7.42, at the interface between the minor binding site (TM1-3,7) and the major binding site (TM3-7) are shown as spheres (black arrowhead: C, white; N, blue; O, red). *p,p*′‑DDT was docked to the minor binding pocket, and the best pose was used for subsequent docking of 16a in the major binding pocket as described in “Methods.” *p < 0.05; **p < 0.01; ***p < 0.001; ****p < 0.0001 for the response in *p,p*′‑DDT–exposed compared with unexposed cells.

The low molecular weight (LMW) agonist, 16a [kindly provided by J. Wrobel (Chemical and Screening Sciences, Wyeth Research, Collegeville, PA), see Figure S2], (Wrobel et al. 2006) can stimulate the FSHR with the same efficiency as FSH through binding to the TMD (Yanofsky et al. 2006). As shown in Figure 3A, increasing concentrations of *p,p*′-DDT potentiated the response to 16a with a 10-fold decrease in the 16a EC_50_ in the presence of 10^–5^ M *p,p*′-DDT.

To analyze putative interactions between *p,p*′-DDT and the TMD, several mutants in helix 3 and in helix 7 (T3.32A, T3.32I, H7.42A, T3.32I-H7.42A) were used. The mutations T3.32I and T3.32A have been identified in women with spontaneous ovarian hyperstimulation syndrome (Montanelli et al. 2004a; Vasseur et al. 2003); they increase the basal activity of the receptor and decrease its ligand specificity (Montanelli et al. 2004b). T3.32, highly conserved in the glycoprotein hormone receptors, is located in the cavity formed by the TMD and can interact with the histidine residue at position 7.42 (Montanelli et al. 2004b). The mutants were expressed at the cell surface, and responsiveness to FSH was unaffected (see Figure S3). Although substitution of T3.32 by alanine maintained the potentiating activity of *p,p*′-DDT on the maximum response induced by FSH (Figure 3B), its substitution by isoleucine abolished this effect (Figure 3C). In addition, *p,p*′-DDT reduced the basal activity of the mutant T3.32I by 30 ± 0.06%. The substitution of H7.42 by alanine reversed the potentiating effect of *p,p*′-DDT to 20 ± 6.28% inhibition (Figure 3D). The basal activity of FSHR H7.42A was unaffected by *p,p*′-DDT. The double mutant T3.32I-H7.42A did not display any sensitivity to *p,p*′-DDT on either the maximal response or the basal activity (Figure 3E). Finally, we evaluated the effects of *p,p*′-DDT on rat FSHR transiently expressed in the CHO cell line. As shown in Figure 3F, an approximately 140% increase in the maximum response without any modification of the EC_50_ (0.04 IU/mL vs. 0.05 IU/mL) was observed. In contrast to the hFSHR response, *p,p*′-DDT induced a significant reduction of the basal activity (30 ± 0.09%) of the rat receptor.

The allosteric effect of *p,p*′-DDT on the activation of FSHR by 16a strongly suggests that both molecules can bind to FSHR. Preliminary models indicated that binding both molecules within the transmembrane cavity required *p,p*′-DDT and 16a in the minor and major binding pockets, respectively (Rosenkilde et al. 2010). The three best-scoring docking poses of *p,p*′-DDT in the minor pocket position showed that one of the *p-*chlorophenyl groups was located in the vicinity of T3.32 and H7.42 (Figure 3G). This observation was consistent with the effects of the mutation of these residues.

### Effects of *p,p*′-DDT on the Specificity of the FSH Receptor

**Figure 4:**
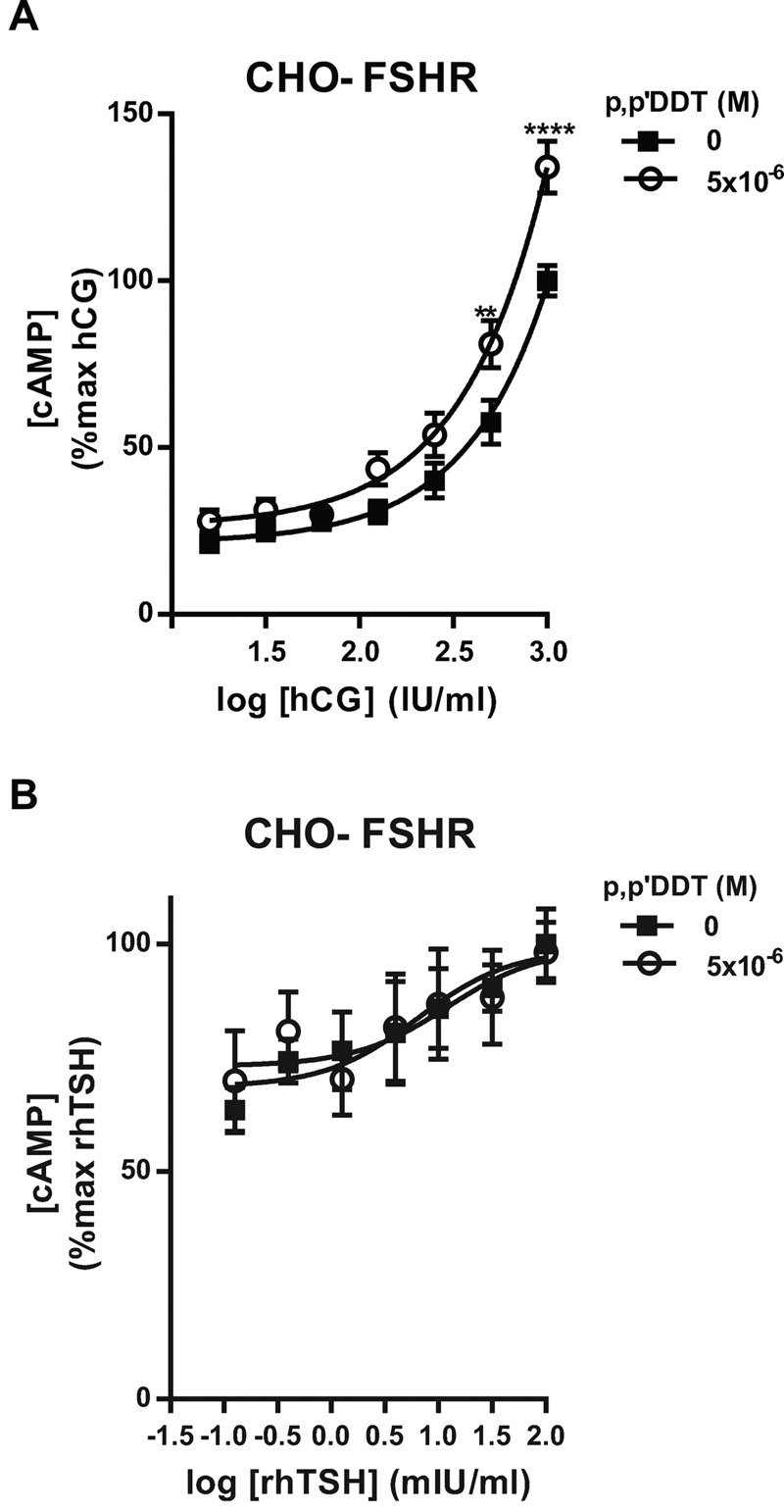
Effects of *p,p*′‑DDT on human chorionic gonadotropin (hCG) and recombinant human thyroid stimulating hormone (rhTSH)–stimulated cAMP production in Chinese hamster ovary–follicle-stimulating hormone receptor (CHO-FSHR) cells. CHO-FSHR cells were stimulated for 30 min with increasing concentrations of hCG or rhTSH with or without 5 **×** 10^–6^ M *p,p*′‑DDT (means ± SEM of three independent experiments performed in triplicate). The maximum response to hCG or rhTSH was arbitrarily set at 100. **p < 0.01, ****p < 0.0001 for the response in *p,p*′‑DDT–exposed compared with unexposed cells, Mann–Whitney U test.

Because some activating mutations, such as T3.32I, of the FSHR TMD make it more responsive to hCG, (De Leener et al. 2006; Montanelli et al. 2004a, 2004b; Smits et al. 2003; Ulloa-Aguirre et al. 2014; Vasseur et al. 2003), the effects of *p,p*′-DDT on the specificity of the FSHR were also analyzed. *p,p*′-DDT enhanced the FSHR response to increasing concentrations of hCG but did not alter the sensitivity of FSHR to thyrotropin (Figure 4), in contrast to the effects of other mutations (T3.32A, T3.32I, H7.42A, T3.32I-H7.42A) (Montanelli et al. 2004b; Vasseur et al. 2003).

### Effects of *p,p*′-DDT–Related Molecules on the FSHR

**Figure 5:**
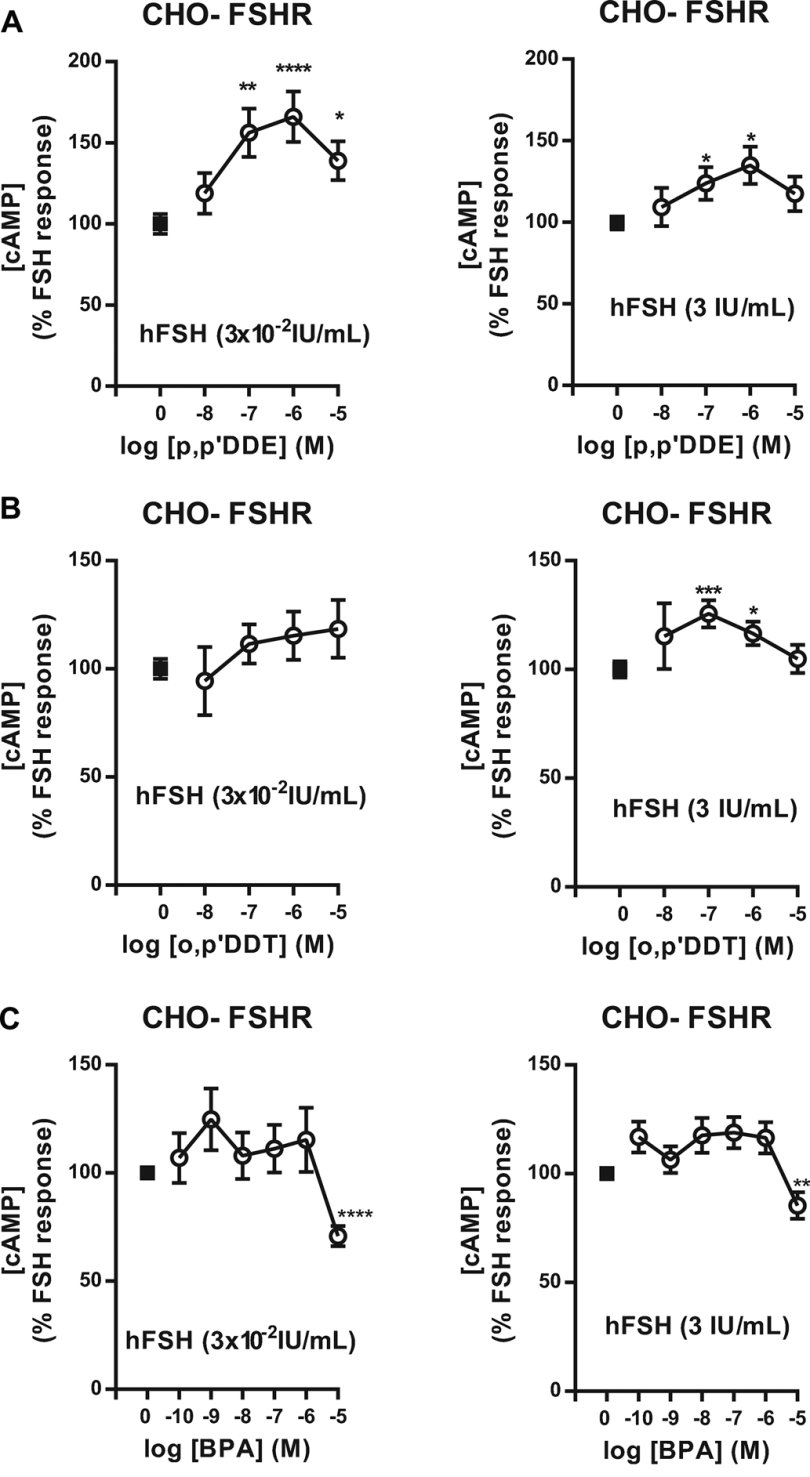
Effects of *p,p*′‑DDE, *o,p*′‑DDT, and bisphenol A (BPA) on follicle stimulating hormone (FSH)-stimulated cAMP production. Chinese hamster ovary–follitropin receptor (CHO-FSHR) cells were stimulated with 3 **×** 10^–2^ IU/mL human FSH (hFSH) (left) and 3 IU/mL hFSH (right) in the presence of increasing doses of *p,p*′‑DDE (*A*), *o,p*′‑DDT (*B*), or BPA (*C*) (means ± SEM of three independent experiments performed in triplicate). The response to hFSH alone was arbitrarily set at 1. *p < 0.05, **p < 0.01, ***p < 0.001, ****p < 0.0001 for the response in *p,p*′‑DDE, *o,p*′‑DDT, or BPA-exposed compared with unexposed cells, Mann-Whitney U test.

*p,p*′-DDT has a biphenolic structure. We hypothesized that other chemicals that are structurally related to *p,p*′-DDT could have similar effects on the FSH-induced cAMP response. *p,p*′-DDT, its metabolite *p,p*′-DDE, and *o,p*′-DDT differ in the number or the position of the chlorine atoms. BPA harbors OH groups instead of chlorine atoms (see Figure S2). The dose–response relationships of *p,p*′-DDE were nonmonotonic for the cAMP accumulation induced by two different doses of hFSH, 0.03 IU/mL and 3 IU/mL. The strongest effects, increases of 66 and 34%, were obtained for 10^–6^ M *p,p*′-DDE (Figure 5A). For *o,p*′-DDT, there were no significant effects on the response to 0.03 IU/mL hFSH, whereas the response to 3 IU/mL hFSH increased by 25% for 10^–7^ M *o,p*′-DDT and was not significant for 10^–5^ M *o,p*′-DDT (Figure 5B). Finally, 10^–5^ M BPA decreased the cAMP production stimulated by FSH 0.3 IU/mL and 3 IU/mL by 30 and 15%, respectively (Figure 5C). We also verified that *p,p*′-DDE, *o,p*′-DDT, and BPA did not induce cell death (see Figure S1).

## Discussion

In the present work, we examined the FSHR as a putative target of *p,p*′-DDT, a known disruptor of reproductive function (Bergman et al. 2013). Previous studies (Bernard et al. 2007; Crellin et al. 1999; Younglai et al. 2004) have shown an alteration of the response of gonadal cells to FSH. Because different molecules and pathways can be affected by *p,p*′-DDT, it was necessary to isolate the FSHR from its native environment, namely sertoli or granulosa cells, to specifically identify disruption of its functions. Therefore, the human FSHR was overexpressed in CHO cells.

We showed that *p,p*′-DDT potentiates the maximum FSH-stimulated cAMP production by the FSHR and thus acts as a positive allosteric modulator. The kinetics of the response to FSH indicate that *p,p*′-DDT acts on the early steps of activation of the FSHR rather than on extinction/prolongation of the signal. Indeed, the effects of *p,p*′-DDT were obvious within 6 min (Figure 1D). Several facts argue for the direct effect of *p,p*′-DDT on the FSHR. The effects of *p,p*′-DDT required the presence of the FSHR because there was no increase in either basal or calcitonin-stimulated cAMP production in untransfected CHO cells. The effect was specific to the FSHR because the closely related LH/hCGR responded differently than the FSHR, with a decrease in the basal activity and no high potentiation of the maximum response. At a concentration of 10^–7^ M, *p,p*′-DDT increased the LH/hCGR maximum response by 8%, whereas 5.10^–6^ M *p,p*′-DDT increased the FSHR maximum response by 32%.

Although these experiments were aimed at investigating the effects of *p,p*′-DDT on FSHR, off-target effects cannot be excluded because the FSHR was studied in a cellular environment. Thus, putative actions of *p,p*′-DDT on the PDE, on AC, and on G protein were examined. The increase in cAMP concentration was not caused by the inhibition of PDEs because *p,p*′-DDT continued to enhance cAMP after inhibition of PDEs by IBMX. The forskolin-induced cAMP production was potentiated only in the presence of the FSHR. This outcome may be an indication of an effect of *p,p*′-DDT on the FSHR even in the absence of FSH. Although no increase in the basal activity of the receptor was detected, this potentiation of the response to forskolin is reminiscent of the effect observed when studying constitutively active mutant GPCRs and is interpreted as an indication of a “pre-activated” state of the GPCR and of the G protein (Alewijnse et al. 1997). Interestingly, potentiation of the response to forskolin was not only caused by the expression of FSHR but also required the presence of *p,p*′-DDT (see Figure S4). Therefore, it can be hypothesized that *p,p*′-DDT binding induces a pre-coupling of FSHR with G_S_, facilitating the activation of AC; this hypothesis requires further investigation.

Several chemicals related to *p,p*′-DDT (*p,p*′-DDE, *o,p*′-DDT and BPA) also affected the FSHR, albeit differently, confirming the specificity of the effects of *p,p*′-DDT. In addition, some mutations of the FSHR in the TMD abolished the effects of *p,p*′-DDT while preserving the response to FSH. This finding suggests that a binding site for the disruptor is located in the TMD.

The electrostatic interactions between ligand and receptor binding pocket play a crucial role in agonist or inverse agonist action (Vezzi et al. 2013). Our results suggest that the chlorine atoms are crucial to the potentiating effect of *p,p*′-DDT on FSHR. This is further illustrated by the inhibiting effect of the 10^–5^ M BPA, which is chlorine-free.

The positive modulation by *p,p*′-DDT was also observed when the FSHR was stimulated by 16a, indicating that both molecules could interact simultaneously with the receptor; this finding was corroborated by molecular docking with *p,p*′-DDT and 16a in the minor and major binding pockets, respectively. The preferred binding pose of *p,p*′-DDT in the minor pocket is consistent with the observed effects of mutation of Thr3.32 and His7.42. The switch from positive to negative allosteric modulation by *p,p*′-DDT upon mutation of H7.42A is reminiscent of an LMW ligand of the thyrotropin receptor whose antagonist effect was reversed to agonist with a point mutation (Hoyer et al. 2013).

The binding of *p,p*′-DDT to the TMD modifies the physicochemical environment of the transmembrane helices of the receptor. This in turn modifies the free energy landscape of the receptor, leading to *p,p*′-DDT acting as a positive allosteric modulator. In addition, the ectodomain of the receptor is proposed to behave as an inhibitor of the TMD (Jiang et al. 2012). The binding of *p,p*′-DDT may also participate in the release of this inhibitory interaction, which may explain the enhanced response to hCG as well.

Other mechanisms may also participate in the allosteric modulation of the FSHR response by *p,p*′-DDT, such as effects on its internalization and desensitization (Krishnamurthy et al. 2003); however, the rapid kinetics of the *p,p*′-DDT effect do not make this likely. The receptor oligomerization (Jiang et al. 2014) may also be affected. Further studies will be necessary to fully understand the mechanisms of the allosteric effects of *p,p*′-DDT. Morover, *p,p*′-DDT can potentially stabilize different conformations of the receptor, thereby leading to biased agonism as described for LMW agonists of the FSHR (Landomiel et al. 2014). It will be interesting to study the impact of *p,p*′-DDT on other signaling pathways.

Several studies indicate that increased activity of the FSH/FSHR pathway (Kumar et al. 1999; Peltoketo et al. 2010), including illegitimate stimulation by hCG (Montanelli et al. 2004a; Smits et al. 2003; Vasseur et al. 2003), may result in adverse effects on reproduction and sexual development. The increased response to FSH in the presence of *p,p*′-DDT that we have shown *in vitro*, and the gain of sensitivity to hCG (and presumably to LH), may therefore be deleterious *in vivo.* Increased stimulation as a result of EDs may contribute to some cases of unexpected and unexplained spontaneous ovarian hyperstimulation syndrome occurring during controlled ovarian stimulation by gonadotropins in assisted reproduction procedures (Jirsová et al. 2010; Machtinger and Orvieto 2014). Whether illegitimate stimulation of FSHR by hCG *in utero* can worsen male and female fetal gonad damage related to *p,p*′-DDT exposure is not known. Our finding that, *in vitro*, *p,p*′-DDT reduced basal activity in the rat FSHR while increasing activity in the human FSHR raises concerns about extrapolating implications of *in vivo* findings from animal models to human health.

## Conclusion

In conclusion, our *in vitro* findings suggest that the human FSHR is a target for *p,p*′-DDT, and they support the potential for effects of *p,p*′-DDT and other EDs on other GPCRs.

## Supplemental Material

(164 KB) PDF
