# Supplementary material for: In Vitro Effects of the Endocrine Disruptor p,p’-DDT on Human Follitropin Receptor
Source: Environ Health Perspect. 2016 Feb 19;124(7):991–9. doi: 10.1289/ehp.1510006 (PMC4937862; doi:10.1289/ehp.1510006)
Supplement: (164 KB) PDF [file ehp.1510006.s001.acco.pdf]

Environ Health Perspect
DOI: 10.1289/ehp.1510006

Note to readers with disabilities: *EHP* strives to ensure that all journal content is accessible to all readers. However, some figures and Supplemental Material published in *EHP* articles may not conform to 508 standards due to the complexity of the information being presented. If you need assistance accessing journal content, please contact ehp508@niehs.nih.gov. Our staff will work with you to assess and meet your accessibility needs within 3 working days.

Supplemental Material

In Vitro Effects of the Endocrine Disruptor p,p'DDT on Human Follitropin Receptor

Mathilde Munier, Julie Grouleff , Louis Gourdin, Mathilde Fauchard, Vanessa Chantreau, Daniel Henrion, Régis Coutant, Birgit Schiøtt, Marie Chabbert, and Patrice Rodien

Table of Contents

Figure S1. Cell survival was measured by an MTT assay. Cells were incubated with p,p'DDT 5.10^{-6}M or p,p'DDE 10^{-5}M or o,p'DDT 10^{-5}M or BPA 10^{-5}M for 30, 60 or 120 min. At the end of the incubation time, MTT (0,5 mg/mL) was added. The formazan coloration at 570 nm was measured. The results are expressed as percentage of viability compared to cells in absence of p,p'DDE or o,p'DDT (means ± SEM of three independent experiments performed in triplicate).

Figure S2. Structure of molecules.

Figure S3. The mutants FSHR T3.32A, T3.32I, H7.42A, T3.32I-H7.42A and the WT transiently expressed in CHO cells. The cell surface expression was determined by flow cytometry. Cells were incubated for 30 min with a 1:10 mouse anti-FSHR antibody. Then they were washed once and incubated for 30 min with a 1:20 FITC-conjugated goat anti-mouse antibody. Flow cytometry measurements were performed on MACSQuant from Miltenyi Biotech (Bergish Gladbach, Deutschland). Expression of mutated receptors is given as a mean of fluorescence intensity (MFI). Cells were stimulated for 30 min with increasing FSH concentrations and the cAMP production measured.

Figure S4. Dose response curve of forskolin (FSK) on CHO or CHO-FSHR cells (means ± SEM of four independent experiments performed in triplicate). The cAMP concentration measured in presence of forskolin on CHO is arbitrarily set at 100%.

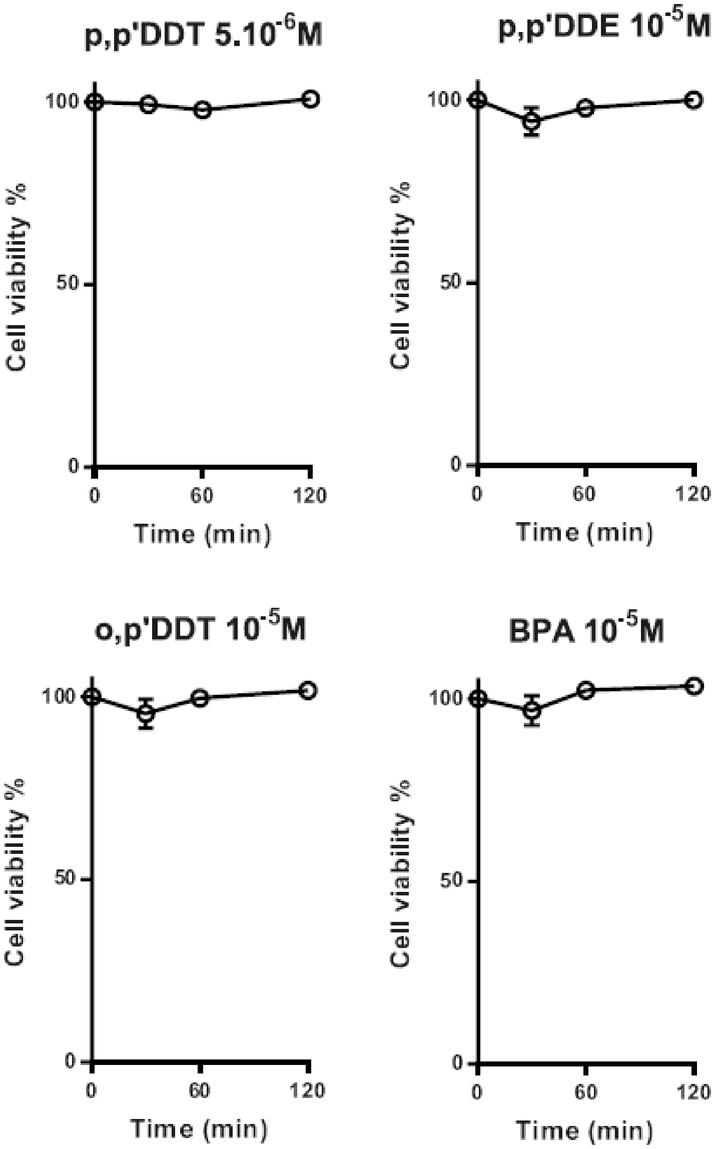

Figure S1

Cell survival was measured by an MTT assay. Cells were incubated with p,p'DDT 5.10^{-6}M or p,p'DDE 10^{-5}M or o,p'DDT 10^{-5}M or BPA 10^{-5}M for 30, 60 or 120 min. At the end of the incubation time, MTT (0,5 mg/mL) was added. The formazan coloration at 570 nm was measured. The results are expressed as percentage of viability compared to cells in absence of p,p'DDE or o,p'DDT (means ± SEM of three independent experiments performed in triplicate).

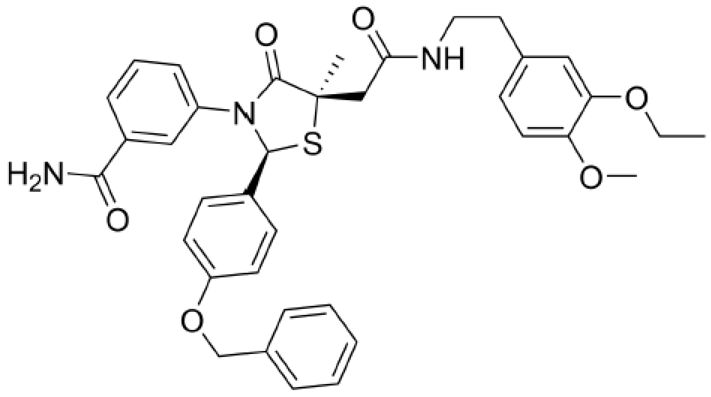

16a

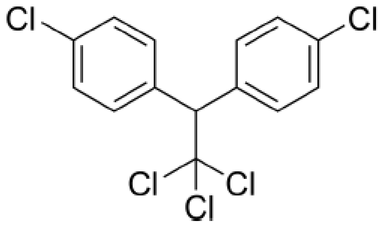

p,p'DDT

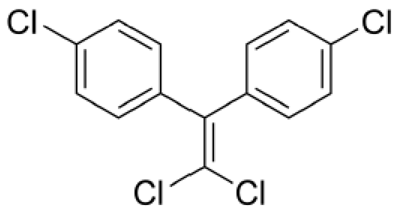

p,p'DDE

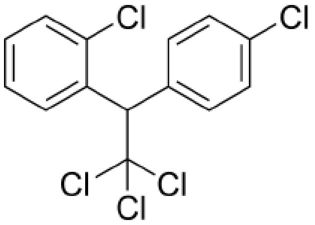

o,p'DDT

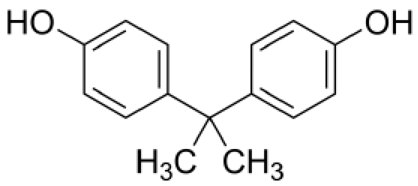

BPA

Figure S2

Structure of molecules

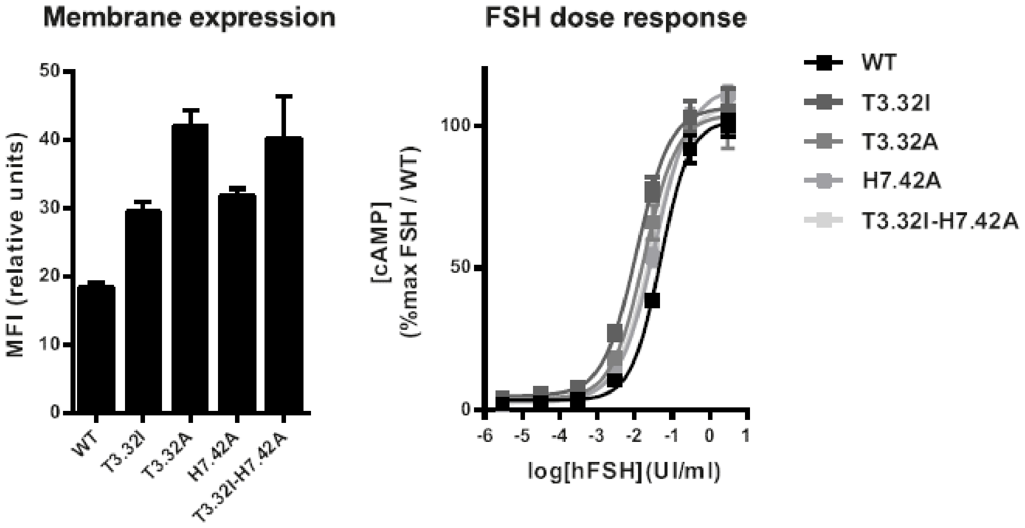

Figure S3

The mutants FSHR T3.32A, T3.32I, H7.42A, T3.32I-H7.42A and the WT transiently expressed in CHO cells. The cell surface expression was determined by flow cytometry. Cells were incubated for 30 min with a 1:10 mouse anti-FSHR antibody. Then they were washed once and incubated for 30 min with a 1:20 FITC-conjugated goat anti-mouse antibody. Flow cytometry measurements were performed on MACSQuant from Miltenyi Biotech (Bergish Gladbach, Deutschland). Expression of mutated receptors is given as a mean of fluorescence intensity (MFI). Cells were stimulated for 30 min with increasing FSH concentrations and the cAMP production measured.

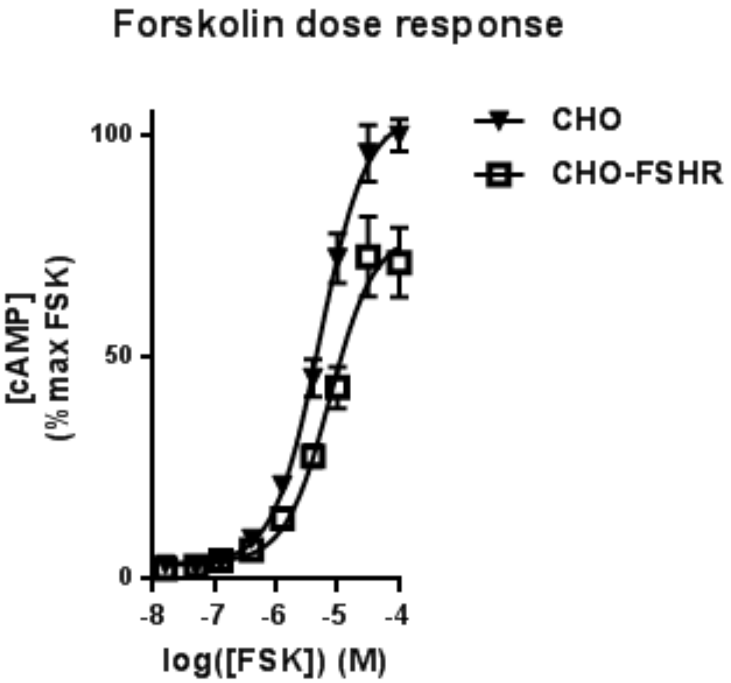

Figure S4

Dose response curve of forskolin (FSK) on CHO or CHO-FSHR cells (means ± SEM of four independent experiments performed in triplicate). The cAMP concentration measured in presence of forskolin on CHO is arbitrarily set at 100%.
